# Florence Nightingale (1820-1910): The Founder of Modern Nursing

**DOI:** 10.7759/cureus.66192

**Published:** 2024-08-05

**Authors:** Yana Turkowski, Victor Turkowski

**Affiliations:** 1 Dermatology Section, Department of Medicine, VA Boston Healthcare System, Boston, USA; 2 Department of Biology, University of Massachusetts, Amherst, USA

**Keywords:** florence nightingale, data analysis, sanitation, medical practice, social reform, healthcare management, nursing

## Abstract

Florence Nightingale, a pioneering figure in the field of nursing during the 19th century, revolutionized medical practices through her innovative approaches to healthcare and dedication to improving patient outcomes. Her advocacy for sanitation significantly reduced mortality rates among patients. Nightingale's pioneering use of data analysis in healthcare and her establishment of nursing education standards laid the foundation for the nursing profession as we know it today. Her contributions continue to resonate in the field of medicine to this day. This paper is a theoretical reflection on Florence Nightingale’s contributions to health and nursing, based on a review of literature from PubMed and Google Scholar databases.

## Introduction and background

Early life

**Figure 1:**
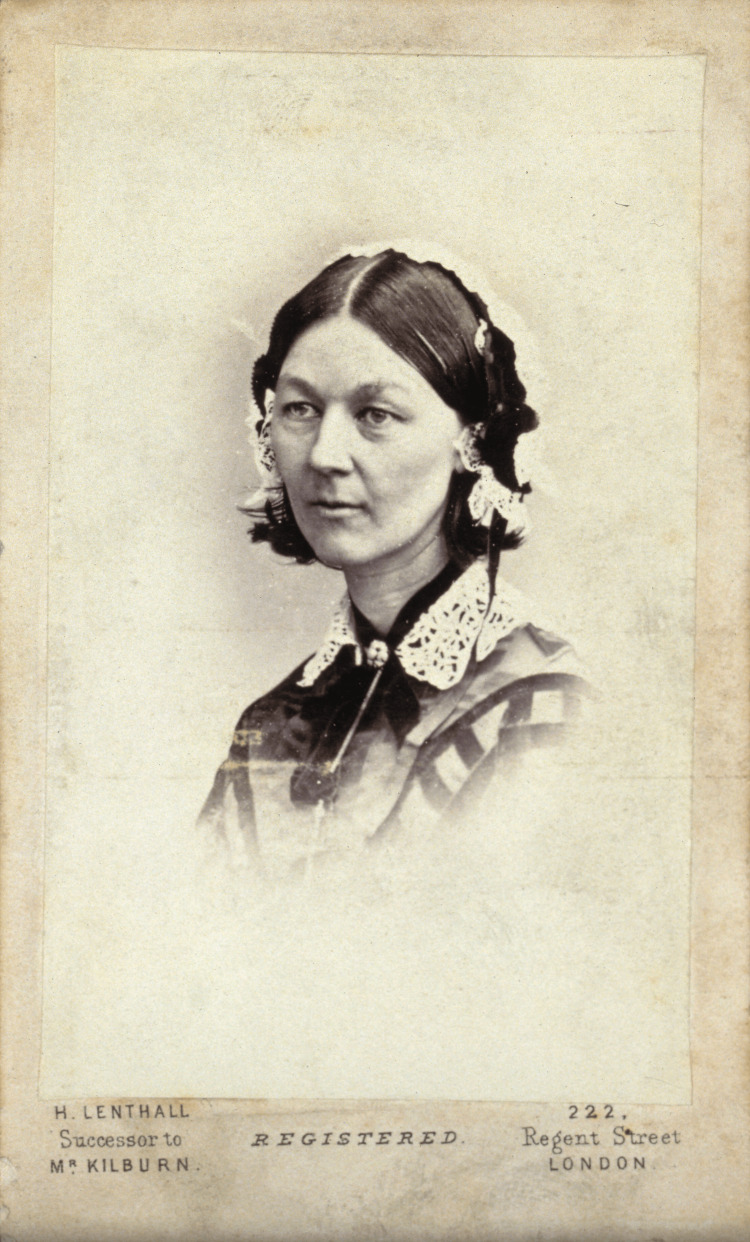
Florence Nightingale (Photograph by H. Lenthall). Credit: Wellcome Collection 13276i. Public Domain Mark.

Florence Nightingale's indelible mark on the landscape of healthcare was forged against the backdrop of the Victorian era, a time characterized by widespread poverty, disease, and inadequate medical care [1] (Figure 1).

Born into a privileged family on May 12, 1820, in Florence, Italy, Nightingale was raised amidst the intellectual and cultural milieu of Victorian England. Despite the societal constraints imposed upon women of her station, she pursued her passion for nursing with unwavering determination, guided by a sense of moral duty and compassion for the suffering of others [2-4].

Florence Nightingale's formative years were marked by a series of transformative experiences that shaped her worldview and fueled her desire for social reform. All the while it is important to note that, in the culture of the time, nursing the sick did not have the noble connotation it has in the modern day. It was considered to be one of the lowliest possible professions when not attached to a religious institution [5]. Nevertheless, encounters with sickness and death within her own family, coupled with her observations of the deplorable conditions in hospitals and workhouses, ignited her fervent commitment to improving the standards of healthcare and sanitation [1]. These experiences laid the foundation for Nightingale's lifelong crusade for healthcare reform and her unwavering dedication to the welfare of the sick and infirm [2].

Crimean War

**Figure 2:**
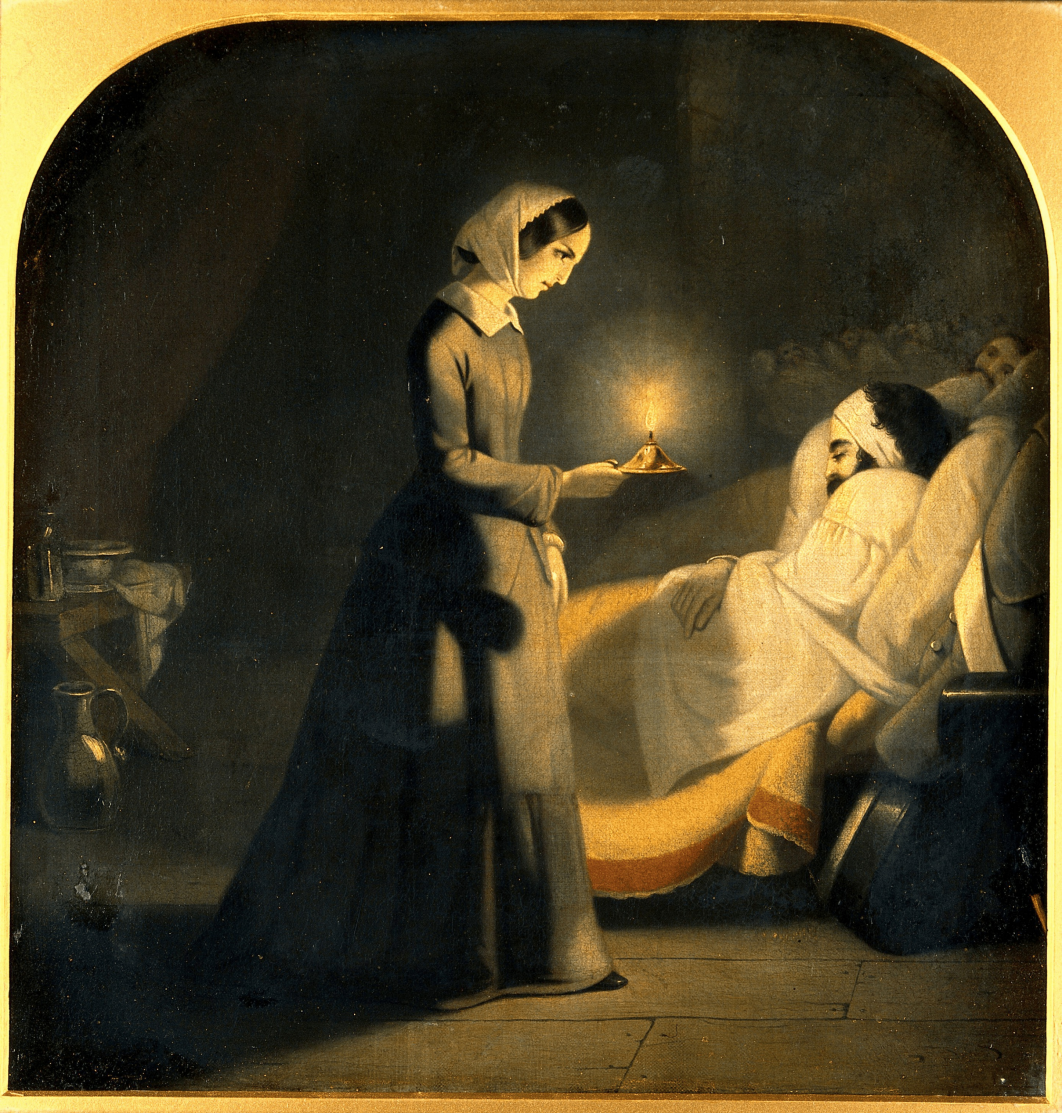
Florence Nightingale as the lady with the lamp (Oil painting attributed to J. Butterworth). Credit: Permission obtained from Wellcome Collection 45755i. Public Domain Mark.

During the Crimean War (1853-1856), Nightingale's humanitarian efforts on the battlefield catapulted her to international prominence and earned her the moniker "The Lady with the Lamp” [6,7] (Figure 2).

**Figure 3:**
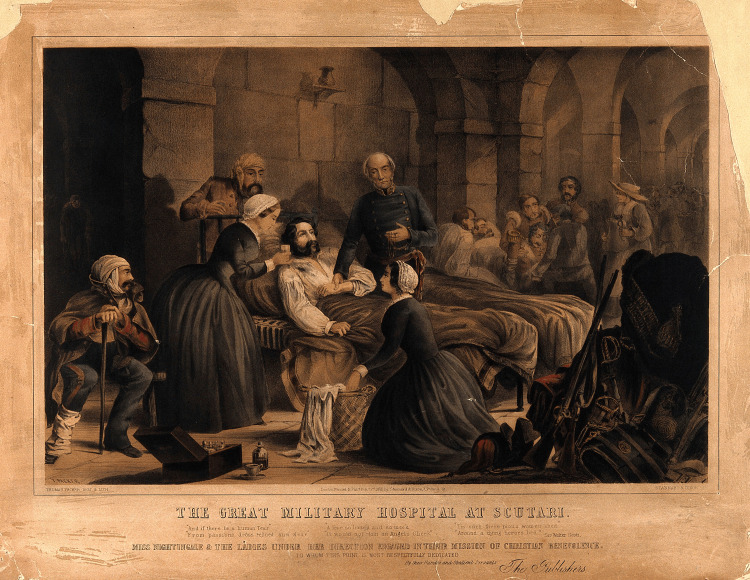
Crimean War: Florence Nightingale and her staff nursing a patient in the military hospital at Scutari (Colored lithograph, c.1855, by T. Packer after himself). Credit: Permission obtained from Wellcome Collection 24565i, Public Domain Mark.

As Nightingale tended to wounded soldiers in the squalid conditions of military hospitals, she witnessed firsthand the devastating toll of infectious diseases and the dire consequences of inadequate medical care [2,4,8] (Figure 3).

Determined to alleviate the suffering of the wounded and improve the dismal state of military healthcare, Nightingale implemented a series of radical reforms that would revolutionize medical practices for generations to come.

## Review

Pioneering contributions to medical services

Florence Nightingale's transformative impact on medical practices is perhaps best understood through a comprehensive review of her pioneering contributions to the fields of nursing, sanitation, and healthcare management. Through her innovative approaches and tireless advocacy, Nightingale left an indelible mark on the landscape of healthcare, revolutionizing medical practices and laying the groundwork for modern healthcare systems.

Central to Nightingale's legacy is her groundbreaking work in sanitation and infection control. She was cognizant of John Snow's pivotal 1854 epidemiological research, which demonstrated a direct connection between cholera and contaminated water from London's Broad Street Pump. This research led Nightingale to recognize the link between the illnesses afflicting her patients and the unclean hospital rooms with unsanitary environments [9]. After identifying the crucial connection between unsanitary conditions and the spread of infectious diseases, Nightingale implemented rigorous hygiene practices that drastically improved the standards of patient care [6].

At the Ottoman Selimiye Barracks, which had been turned into a temporary military hospital, Nightingale reorganized and refined its sanitation over the course of the Crimean War [10]. Her emphasis on cleanliness, ventilation, and the maintenance of sanitary environments within healthcare facilities led to dramatic reductions in mortality rates and laid the foundation for modern infection control protocols [6]. Nightingale's advocacy for sanitary reforms remains one of the most important public health initiatives to this day, underscoring the enduring relevance of her contributions [1].

Pioneering statistical analysis in medicine

Drawing on her background in mathematics and statistics, Nightingale collected and analyzed data on patient outcomes, mortality rates, and healthcare practices [1]. Her statistical analyses provided critical insights into the effectiveness of medical interventions and the impact of environmental factors on patient health. Nightingale's use of statistical analysis is notably shown in her work during the Crimean War. In her seminal report titled "Notes on Matters Affecting the Health, Efficiency, and Hospital Administration of the British Army," Nightingale presented statistical data on mortality rates among wounded soldiers in military hospitals. Specifically, through careful analysis of this data, Nightingale demonstrated that the majority of deaths were not due to battlefield injuries, but rather to preventable diseases such as typhus, cholera, and dysentery [11-13].

Nightingale's statistical analyses revealed the profound impact of unsanitary conditions on patient outcomes, bringing attention to the urgent need for improved sanitation practices in healthcare facilities [14]. Acting on her findings, Nightingale implemented rigorous hygiene measures: the provision of clean water, proper ventilation, and the thorough cleaning of hospital wards. These interventions led to a significant reduction in mortality rates and laid the foundation for modern infection control protocols [6]. However, Nightingale's use of statistical analysis did not stop at the battlefield. Throughout her career, she collected and analyzed data sets on a wide range of healthcare issues, including hospital management, nursing practices, and public health. Her innovative approach to data analysis paved the way for evidence-based medicine and epidemiology, emphasizing the importance of empirical evidence in guiding medical decision-making [1]. Her pioneering work laid the foundation for evidence-based nursing and underscored the importance of data-driven decision-making in improving patient outcomes. These initial, revolutionary efforts to reconcile knowledge and practices to reduce harm, both physical and psychological, continue to be timeless in their relevance, to the present day, as recovery efforts continue against the COVID-19 pandemic [15].

Establishment of the nursing profession and education

**Figure 4:**
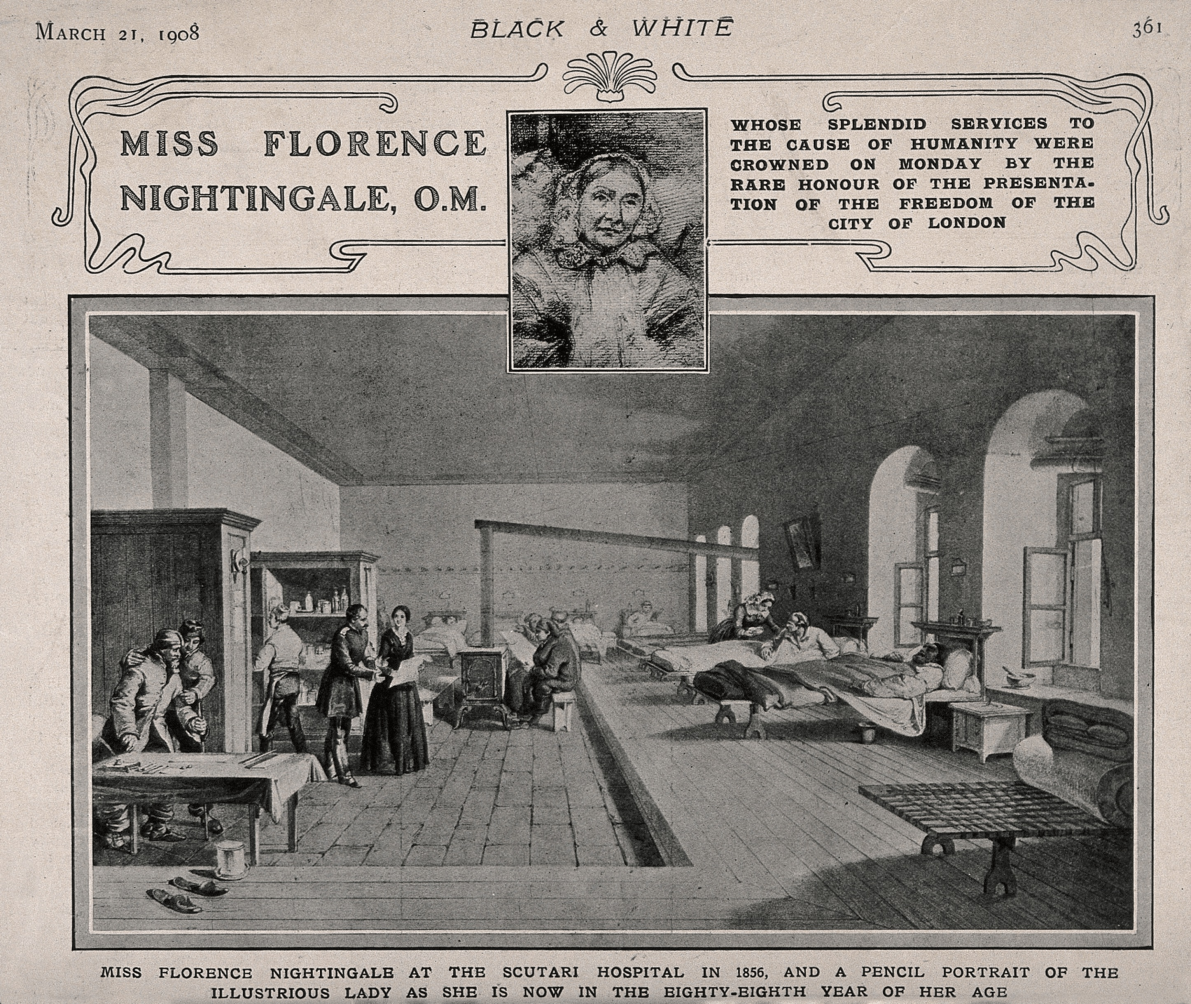
Crimean War: Florence Nightingale at Scutari Hospital, 1856, plus a portrait drawing (Process print, 1908). Crimean War: Florence Nightingale at Scutari Hospital, 1856, plus a portrait drawing. Process print, 1908.

In addition to her work in sanitation, Nightingale played a pivotal role in professionalizing the nursing profession and establishing standards for nursing education [16,17]. The world's first secular nursing school at St. Thomas' Hospital in London was founded by Nightingale in 1860. The establishment of this school was a pivotal moment in the history of nursing education and professionalization. Florence Nightingale recognized the urgent need for systematic training and education for nurses, as she believed that competent and compassionate nursing care was essential for improving patient outcomes. The Nightingale Training School for Nurses was founded on the principles of discipline, education, and compassion. Nightingale envisioned a rigorous curriculum that would provide nurses with both theoretical knowledge and practical skills, ensuring that they were well-prepared to meet the demands of their profession. The curriculum included instruction in anatomy, physiology, hygiene, and nursing care techniques, as well as practical experience working with patients under the supervision of experienced nurses [2,17,18]. Nightingale was closely involved in the development and administration of the nursing school, personally selecting the staff and overseeing the curriculum. She was committed to maintaining high standards of education and professionalism, and she worked tirelessly to ensure that the nursing school lived up to her vision [6,18] (Figure 4).

The Nightingale Training School for Nurses quickly became a model for nursing education worldwide, and it played a crucial role in elevating the status of nursing as a respected profession. Graduates of the school went on to work in hospitals and healthcare settings around the world, carrying Nightingale's principles of compassionate care and scientific rigor with them [3,18].

Modern impact

In addition to her work at the Nightingale Training School for Nurses, Florence Nightingale wrote extensively on nursing education and training, publishing influential studies such as *Notes on Nursing: What It Is, and What It Is Not* [11]. In this book, Nightingale gives the timeless advice, “Every nurse ought to be careful to wash her hands very frequently during the day” [11]. This emphasis on hand hygiene is one of Nightingale's lasting legacies and has permeated much of the world. This now-common practice drastically decreases the risk of infectious diseases in day-to-day activities, with its importance increasing exponentially in the healthcare setting. It is from these simple gestures and practices that a holistic and healthy approach to patient care is built. Through her writings and educational efforts, Nightingale laid the foundation for modern nursing education and helped shape the development of the nursing profession.

## Conclusions

Florence Nightingale's enduring legacy continues to profoundly influence medicine and nursing today. Her innovative approaches to sanitation laid the groundwork for global health standards, significantly reducing the spread of infectious diseases. The establishment of rigorous nursing education standards has elevated the profession, ensuring high-quality care worldwide. Moreover, her pioneering use of statistical analysis has become integral to evidence-based healthcare practices, guiding modern medical decisions and policy formulation. Nightingale's visionary initiatives remain pivotal in shaping the contemporary landscape of healthcare, advancing patient outcomes and healthcare efficiency across the globe.
